# Association between uric acid to lymphocyte ratio and poor functional outcomes in acute ischemic stroke patients

**DOI:** 10.3389/fneur.2026.1825152

**Published:** 2026-05-28

**Authors:** Xu Zhu, Yijun Zhang, Guoyuan Yu, Anxin Wang, Xiaoli Zhang, Shifeng Xiang, Xia Meng, Yiping Wu

**Affiliations:** 1 Department of Neurology, Handan Central Hospital, Handan, China; 2 Department of Neurology, Beijing Tiantan Hospital, Capital Medical University, Beijing, China; 3 China National Clinical Research Center for Neurological Diseases, Beijing Tiantan Hospital, Capital Medical University, Beijing, China; 4 Department of Epidemiology, Beijing Neurosurgical Institute, Beijing Tiantan Hospital, Capital Medical University, Beijing, China; 5 Department of Clinical Epidemiology and Clinical Trial, Capital Medical University, Beijing, China; 6 Beijing Municipal Key Laboratory of Clinical Epidemiology, Beijing, China

**Keywords:** acute ischemic stroke, functional outcome, inflammation, stroke, uric acid to lymphocyte ratio

## Abstract

**Background:**

Inflammation has an important impact on the pathological progression associated with ischemic stroke. Serum uric acid (UA) to lymphocyte ratio (ULR) is a biomarker that responds to the level of inflammation but is not definitively associated with the clinical outcomes in patients with acute ischemic stroke (AIS).

**Methods:**

The data were obtained from the Third China National Stroke Registry (CNSR-III). Enrolled AIS patients were grouped by ULR quartiles at admission. The outcomes were poor functional outcomes (modified Rankin Scale [mRS] score of 3–6 or 2–6) and all-cause mortality at 3 months and 1 year. The associations of ULR with the risk of poor functional outcome and all-cause mortality were analyzed by multivariable logistic regression and Cox proportional hazards regression.

**Results:**

A total of 8,241 patients were included from the CNSR-III study. After adjusting for confounders, it was found that patients in the highest ULR quartile had higher mRS scores of 2–6 (odds ratio [OR], 1.33; 95% confidence interval [CI], 1.15–1.53) and 3–6 (OR, 1.35; 95% CI, 1.16–1.57) at the 3-month follow-up. Additionally, the highest ULR quartile was associated with an increased risk of all-cause mortality at the 3-month follow-up (hazard ratio [HR], 1.97; 95%CI, 1.22–3.18). Similar results were observed at the 1-year follow-up.

**Conclusion:**

Elevated ULR increased the risks of poorer functional outcomes and all-cause mortality in the AIS patients. However, this observational study was limited by potential unmeasured confounders, selection bias, residual confounding, and restricted generalizability to other populations.

## 1 Introduction

Acute ischemic stroke (AIS), mainly caused by atherosclerosis, is increasing in incidence and associated with disability and death, especially in East Asian countries (1). Evidence increasingly suggests that inflammation is involved in all stages of the pathologic process of AIS onset, development, and regression. Ischemic injury rapidly triggers systemic inflammatory cascade and excessive oxidative stress, which synergistically exacerbate blood–brain barrier disruption, neuronal apoptosis, and brain tissue damage. Inflammatory responses and neuron necrosis are key factors influencing stroke severity and poor outcomes (2–4). Systemic inflammation and oxidative stress are pathological drivers of AIS progression and prognosis, reliable biomarkers that sensitively reflect both processes are urgently needed for risk stratification and prognostic evaluation.

Uric acid (UA) is a significant risk factor for inducing vascular inflammation (5). UA induces various pro-inflammatory and pro-oxidative effects in vascular cells leading to endothelial cell dysfunction. High levels of UA amplify oxidative stress injury and systemic inflammatory responses in AIS by disrupting endothelial homeostasis, promoting leukocyte adhesion and infiltration, and aggravating cerebral microcirculation disturbance (6). Elevated UA levels have been increasingly demonstrated to be closely associated with oxidative stress, inflammatory responses, and cardiovascular and neurological diseases (7–9). As an easily accessible routine laboratory indicator in emergency practice, serum uric acid is also closely correlated with acute neuroinflammation, oxidative stress imbalance and cerebral microcirculatory dysfunction, and has emerged as a valuable auxiliary biomarker for early risk stratification and prognostic evaluation among acute ischemic stroke patients (10, 11). Dysregulated uric acid metabolism not only mirrors systemic metabolic disturbance but also actively participates in the entire pathological cascade of ischemic brain injury, further explaining its broad clinical relevance in acute stroke management (12).

A decrease in lymphocytes (LY) characterizes the systemic inflammatory response, reflecting damage to the lymphatic system and general poor physical condition. In the acute phase of AIS, excessive systemic inflammation and oxidative stress induce lymphocyte apoptosis, redistribution, and consumption, resulting in reduced LY counts and this reduction reflects the intensity of the systemic inflammatory response and the degree of immune dysfunction (13). A compromised immune system contributes to a cascading response to stroke. The neutrophil-lymphocyte ratio, a widely studied biomarker, best demonstrates a negative relationship between LY and ischemic stroke. Clinical studies and meta-analyses have shown that the higher neutrophil-lymphocyte ratio is associated with a poorer prognosis in AIS (14–16). However, the role of UA and LY in stroke remains controversial at the relationship with poor prognosis in AIS patients (17). In the specific pathological context of AIS, single biomarkers (UA or LY) can only partially reflect either oxidative stress or immune-inflammatory status, lacking comprehensiveness. In contrast, the UA-to-LY ratio (ULR) integrates the dual pathological dimensions of UA-mediated oxidative stress and systemic inflammation, and LY-represented immune-inflammatory suppression. The combined effect of UA and LY, has emerged as a novel risk prediction tool for systemic inflammatory conditions and has been widely used in the evaluation of tumors and cardiovascular diseases (18, 19). However, whether ULR could have a better predictive value as a new inflammatory biomarker for the prognosis of AIS has not yet been investigated.

Therefore, the present study attempted to assess the associations between ULR with poor functional prognosis and all-cause mortality in AIS patients based on a large multicenter-based case study.

## 2 Methods

### 2.1 Study design and population

The third China National Stroke Registry (CNSR-III) is a national registry and a detailed study design has been published (20). From August 2015 to March 2018, a total of 15,166 patients with AIS or transient ischemic attack (TIA) were enrolled in 201 hospitals as study subjects. All enrolled patients aged 18 years or above were diagnosed within 7 days. This research was conducted in strict compliance with the Declaration of Helsinki and received approval from the Ethics Committee of Beijing Tiantan Hospital (IRB approval number: KY2015-001-01).

### 2.2 Baseline data collection

Research coordinators at each site proactively collected baseline data through face-to-face interviews or by reviewing medical records. These data included age, gender, marital status, education level, heavy drinking, current smoking, body mass index (BMI, calculated as weight in kilograms divided by the square of height in meters, kg/m^2^), systolic blood pressure (SBP), heart rate at admission, National Institutes of Health Stroke Scale (NIHSS) score at admission, medical history (IS or TIA, intracerebral hemorrhage [ICH], hypertension, coronary heart disease, dyslipidemia), the cause of AIS classified according to the Trial of Org 10,172 in Acute Stroke Treatment (TOAST) criteria, in-hospital treatment (antiplatelet, anticoagulant, antihypertensive, antihyperlipidemic, hypoglycemic), laboratory test (albumin [ALB], Serum total cholesterol [TC], Serum triglyceride [TG]).

### 2.3 Sample collection and definition of ULR

Fasting blood samples were obtained within 24 h of admission following stroke onset. ULR was calculated as UA (mg/dL)/lymphocyte count (×10^9^/L). ULR levels were categorized into four quartile-based groups: <2.13, 2.13–2.87, 2.87–3.94, and >3.94.

### 2.4 Follow-up and outcome assessment

Patients were followed up at 3 months and 1 year after onset by trained study coordinators through face-to-face or telephone interviews. Functional outcomes were defined using the modified Rankin Scale (mRS) score which is a measure of disability widely used to assess recovery after stroke. The primary outcomes were mRS 3–6 at 3 months and 1 year, and the secondary outcomes were mRS 2–6 and all-cause mortality at 3 months and 1 year. All-cause mortality included deaths from any cause. Mortality information was obtained from relatives and verified through death certificates from attended hospitals or local civil registries.

### 2.5 Statistical analysis

Patients were divided into four groups by ULR quartiles. Continuous variables were presented as medians and interquartile ranges (IQR) and compared using the Kruskal-Wallis test. Categorical variables were expressed as percentages and compared using the chi-square test or Fisher’s exact test. For poor functional outcomes mRS 2–6 and mRS 3–6, logistic regression models were used to calculate the odds ratio (OR) and 95% CI. Hazard ratios (HR) and 95% confidence intervals (CI) for all-cause mortality were calculated using Cox proportional risk models. Variables adjusted in multivariable models were age and gender, education, SBP, drinking and smoking, TOAST, medical history (IS or TIA, ICH, hypertension, coronary heart disease, dyslipidemia), in-hospital treatment (antiplatelet, anticoagulant, antihypertensive, antihyperlipidemic, hypoglycemic), and laboratory test (ALB, TC, TG). Considering median ULR in each group as a continuous variable to perform the linear trend tests.

Restricted cubic splines (RCS) were used to explore relationships between continuous ULR with the risks of poor functional outcomes and all-cause mortality. Subgroup analyses were performed based on age (<65 or ≥65 years), sex (male or female), BMI (≤25 or >25 kg/m^2^), and current smoking (yes or no). Additionally, we used C statistics, integrated discrimination improvement (IDI), and net reclassification index (NRI) to evaluate the predictive performance of ULR, LY, and UA beyond the basic model.

A two-sided *p* value of less than 0.05 was considered significant. All statistical analyses were performed using SAS 9.4 software (SAS Institute, Inc., Cary, NC).

## 3 Results

### 3.1 Baseline characteristics

**Figure 1:**
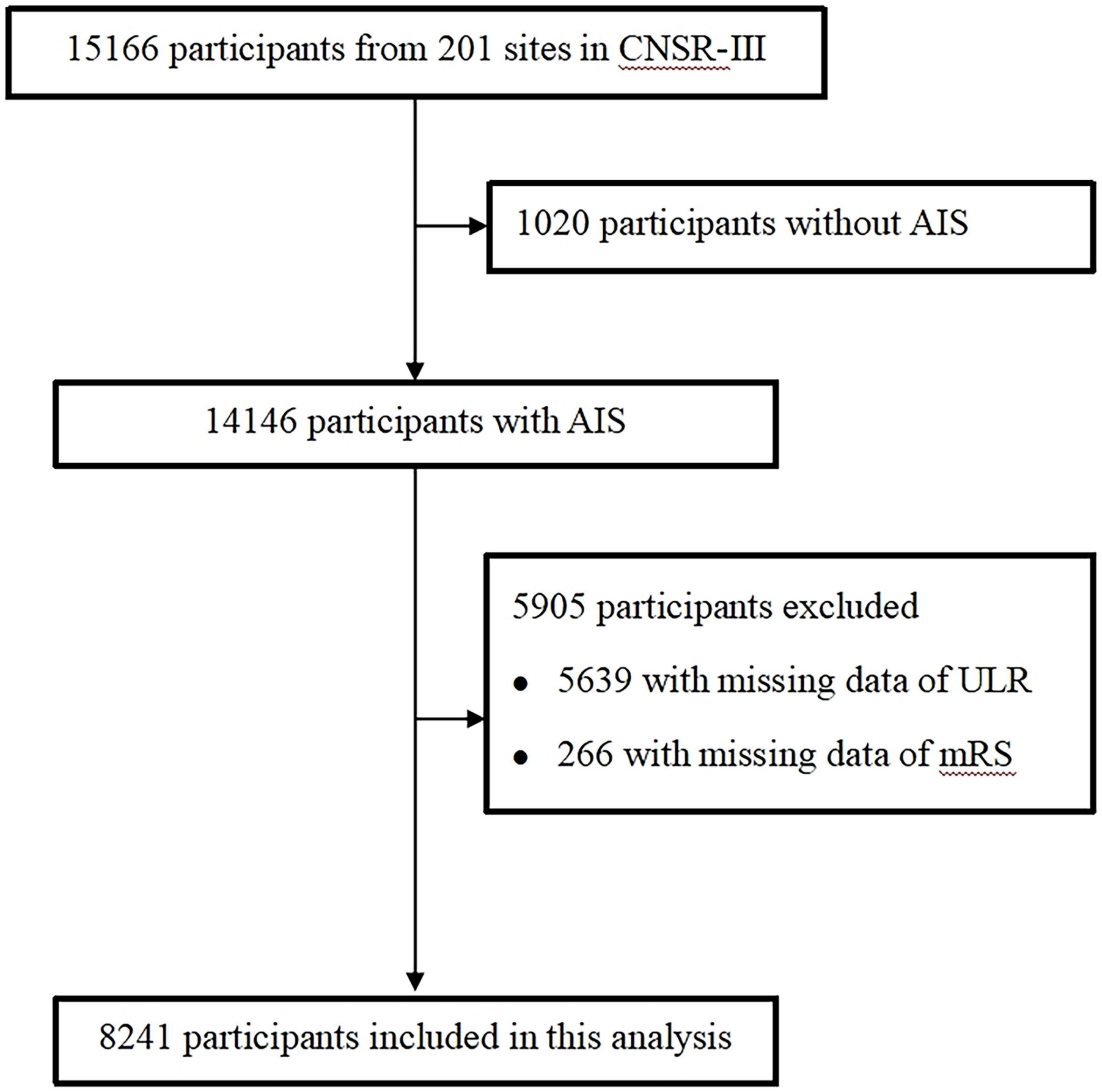
Flow chart of sample selection and the exclusion criteria. CNSR-III, the Third China National Stroke Registry; AIS, acute ischemic stroke; ULR, lymphocyte ratio.

Among the 15,166 patients enrolled in the CNSR-III study, 1,020 patients diagnosed with TIA were excluded, 5,639 patients were also excluded due to lack of ULR data as well as 266 patients who were lost to follow-up. Ultimately, we included 8,241 patients in this study (Figure 1). Table 1 shows the baseline characteristics of the different groups of participants. Patients with higher ULR levels tended to be older, male, with lower BMI, higher NIHSS scores, heavy drinking, and lower TG levels.

**Table 1:** Baseline characteristics of patients according to ULR quartiles.

Variable	Total	Serum ULR				*p* value
		Q1 (<2.13)	Q2 (2.13–2.87)	Q3 (2.87–3.94)	Q4 (>3.94)	
Patients	8,241	2060	2060	2061	2060	
Age, years, median (IQR)	62 (54–70)	61 (54–68)	61 (54–69)	62 (54–70)	65 (57–73)	<0.001
Male, *n* (%)	5,671 (68.81)	1,139 (55.29)	1,400 (67.96)	1,516 (73.56)	1,616 (78.45)	<0.001
Marital, *n* (%)						0.926
Married	7,762 (94.19)	1936 (93.98)	1946 (94.47)	1940 (94.13)	1940 (94.17)	
Others	479 (5.81)	124 (6.02)	114 (5.53)	121 (5.87)	120 (5.83)	
Education, *n* (%)						0.032
Middle school or below	7,535 (91.43)	1910 (92.72)	1891 (91.80)	1861 (90.30)	1873 (90.92)	
High school or above	706 (8.57)	150 (7.28)	169 (8.20)	200 (9.70)	187 (9.08)	
BMI, kg/m^2^, median (IQR)	24.49 (22.58–26.57)	24.44 (22.59–26.35)	24.49 (22.53–26.57)	24.61 (22.86–26.81)	24.34 (22.49–26.42)	0.032
SBP, mmHg, median (IQR)	149 (135–164.5)	149.5 (135–163.5)	148.5 (135–163.5)	148.5 (135–166)	149.5 (135.5–165)	0.647
Heart rate, median (IQR)	76 (68–80)	76 (70–82)	76 (68–80)	75 (68–80)	75 (68–80)	0.069
NIHSS score, median (IQR)	3 (2–6)	3 (1–6)	3 (2–6)	3 (2–6)	4 (2–6)	<0.001
Heavy drinking, *n* (%)	1,165 (14.14)	241 (11.70)	293 (14.22)	308 (14.94)	323 (15.68)	0.002
Current smoking, *n* (%)	2,649 (32.14)	659 (31.99)	749 (36.36)	655 (31.78)	586 (28.45)	<0.001
Medical history, *n* (%)						
IS or TIA	1886 (22.89)	462 (22.43)	454 (22.04)	474 (23.00)	496 (24.08)	0.431
ICH	150 (1.82)	27 (1.31)	35 (1.70)	51 (2.47)	37 (1.80)	0.044
Hypertension	5,199 (63.09)	1,276 (61.94)	1,276 (61.94)	1,299 (63.03)	1,348 (65.44)	0.066
Coronary heart disease	875 (10.62)	210 (10.19)	214 (10.39)	217 (10.53)	234 (11.36)	0.634
Dyslipidemia	666 (8.08)	157 (7.62)	167 (8.11)	178 (8.64)	164 (7.96)	0.686
TOAST types, *n* (%)						<0.001
LAA	2091 (25.37)	537 (26.07)	542 (26.31)	515 (24.99)	497 (24.13)	
CE	546 (6.63)	108 (5.24)	110 (5.34)	134 (6.50)	194 (9.42)	
SAO	1824 (22.13)	471 (22.86)	464 (22.52)	476 (23.10)	413 (20.05)	
Others	3,780 (45.87)	944 (45.83)	944 (45.83)	936 (45.41)	956 (46.41)	
In-hospital treatment, *n* (%)						
Antiplatelet	7,946 (96.42)	1984 (96.31)	1993 (96.75)	1999 (96.99)	1970 (95.63)	0.096
Anticoagulant	821 (9.96)	194 (9.42)	184 (8.93)	195 (9.46)	248 (12.04)	0.004
Antihypertensive	3,865 (46.90)	913 (44.32)	936 (45.44)	996 (48.33)	1,020 (49.51)	0.002
Antihyperlipidem	7,938 (96.32)	1988 (96.50)	1975 (95.87)	1993 (96.70)	1982 (96.21)	0519
Hypoglycemic	2,157 (26.17)	703 (34.13)	537 (26.07)	492 (23.87)	425 (20.63)	<0.001
IVT	488 (5.92)	118 (5.73)	94 (4.56)	160 (7.76)	116 (5.63)	0.013
EVT	21 (0.25)	9 (0.44)	3 (0.15)	9 (0.44)	0 (0.00)	0.276
Laboratory test						
ALB, g/L, median (IQR)	40.40 (37.9–43.00)	40.40 (38.00–42.95)	40.50 (38.00–42.90)	40.40 (38.00–43.10)	40.10 (37.70–43.00)	0.118
TG, g/L, median (IQR)	1.35 (1.02–1.85)	1.36 (1.03–1.88)	1.36 (1.03–1.91)	1.36 (1.025–1.85)	1.30 (0.98–1.81)	0.005
TC, g/L, median (IQR)	3.97 (3.31–4.71)	4.00 (3.33–4.725)	3.95 (3.31–4.69)	3.95 (3.34–4.7)	3.95 (3.27–4.74)	0.735

ULR, uric acid to lymphocyte ratio; SD, standard deviation; IQR, interquartile range; BMI, body mass index; SBP, systolic blood pressure; NIHSS, National Institute of Health Stroke Scale; mRS, modified Rankin Scale; IS, ischemic stroke; TIA, transient ischemic attack; ICH, intracranial hemorrhage; TOAST, the Trail of Org 10,172 in Acute Stroke Treatment; LAA, large artery atherosclerosis; CE, cardioembolism; SAO, small artery occlusion; IVT, intravenous thrombolysis; EVT, endovascular thrombectomy; ALB, albumin; TC, Serum total cholesterol; TG, Serum triglyceride.

### 3.2 Association of ULR with clinical outcomes

**Figure 2:**
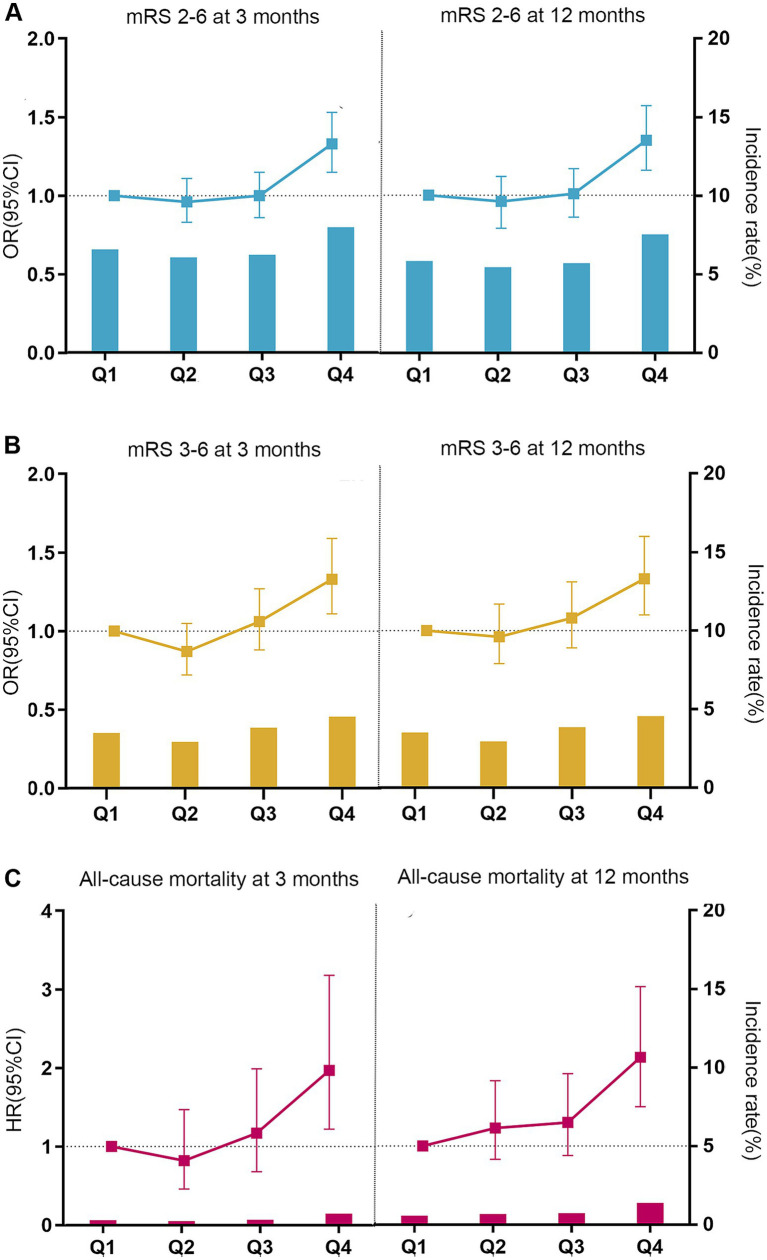
Incidence rate and HR or OR of poor outcomes and all-cause mortality at 3 months and 1 year. **(A)** Incidence rate and OR distribution of the outcome mRS 2–6 at 3 months and 12 months; **(B)** incidence rate and OR distribution of the outcome mRS 3–6 at 3 months and 12 months; **(C)** incidence rate and HR distribution of the all-cause mortality at 3 months and 12 months. Adjusted for age, gender, education, SBP, drinking and smoking, TOAST, medical history (IS or TIA, ICH, hypertension, coronary heart disease, dyslipidemia), in-hospital treatment (antiplatelet, anticoagulant, antihypertensive, antihyperlipidemic, hypoglycemic), and laboratory test (ALB, TC, TG). SBP, systolic blood pressure; mRS, modified Rankin Scale; IS, ischemic stroke; TIA, transient ischemic attack; ICH, intracranial hemorrhage; TOAST, the Trail of Org 10,172 in Acute Stroke Treatment; ALB, albumin; TC, Serum total cholesterol; TG, erum triglyceride; HR, hazard ratio; OR, odds ratio.

**Figure 3:**
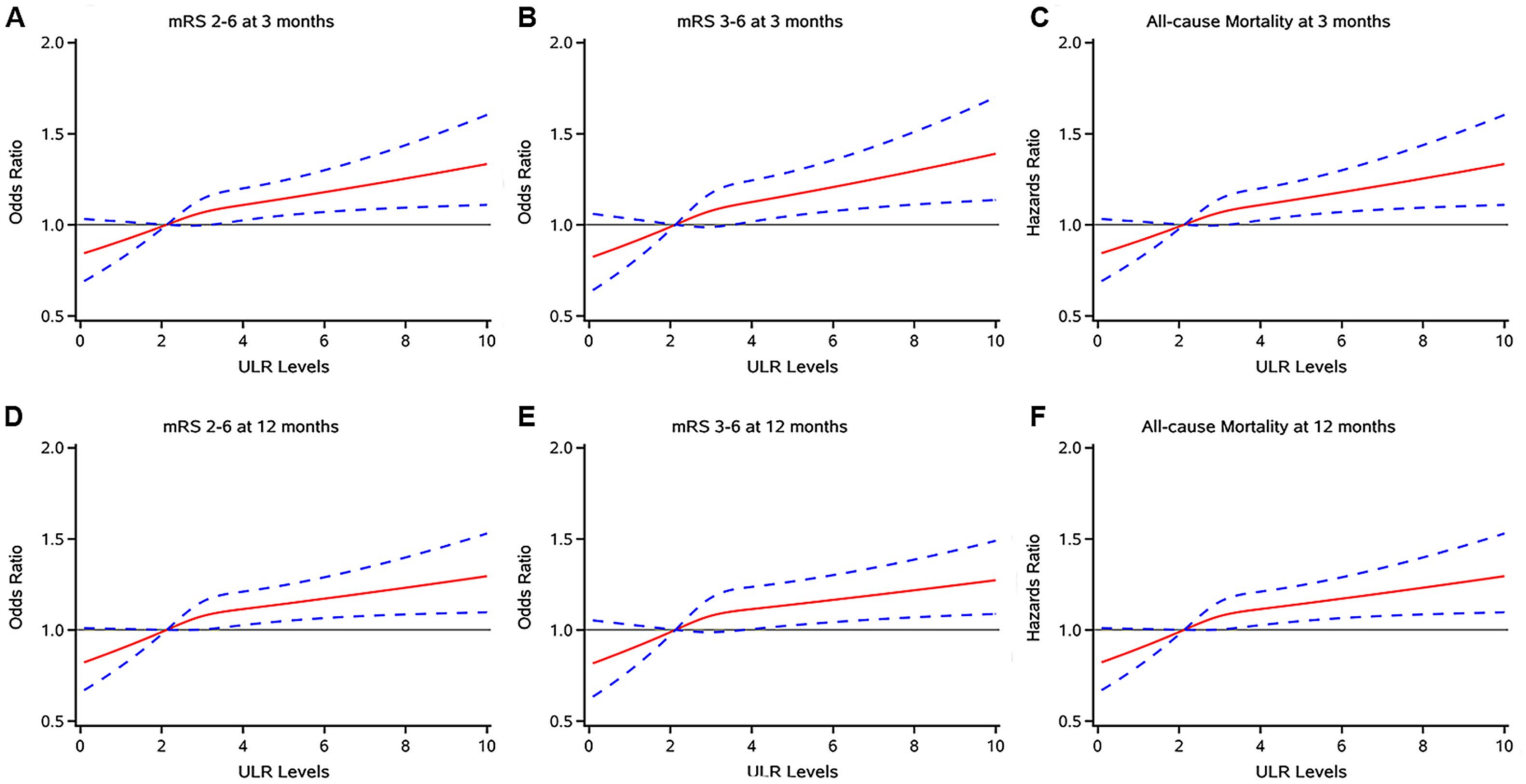
Restricted cubic splines showed the association between ULR and clinical outcomes at 3 months and 12 months. The red line indicates adjusted OR/HR, and the blue lines indicate the 95% CI. **(A–C)** The clinical outcomes include mRS and all-cause mortality at 3 months; **(D–F)** the clinical outcomes at 12 months. Adjusted for age, gender, education, SBP, drinking and smoking, TOAST, medical history (IS or TIA, ICH, hypertension, coronary heart disease, dyslipidemia), in-hospital treatment (antiplatelet, anticoagulant, antihypertensive, antihyperlipidemic, hypoglycemic), and laboratory test (ALB, TC, TG). SBP, systolic blood pressure; mRS, modified Rankin Scale; IS, ischemic stroke; TIA, transient ischemic attack; ICH, intracranial hemorrhage; TOAST, the Trail of Org 10,172 in Acute Stroke Treatment; ALB, albumin; TC, erum total cholesterol; TG, erum triglyceride; HR, hazard ratio; OR, odds ratio; ULR, uric acid to lymphocyte ratio.

**Figure 4:**
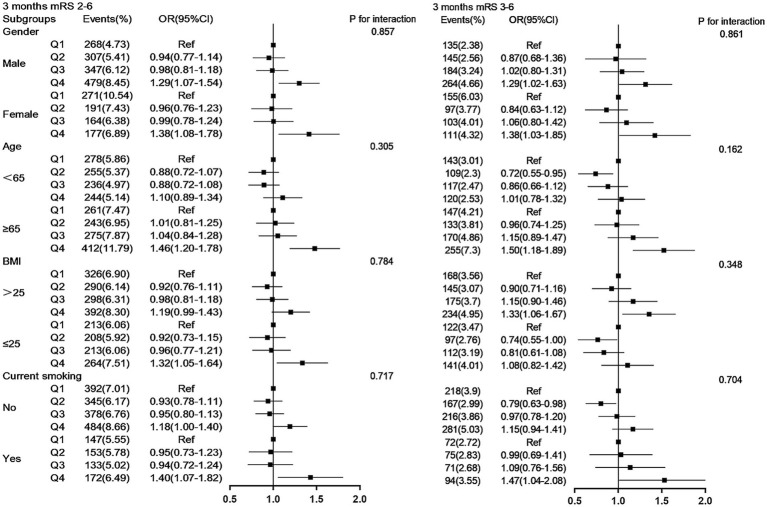
Subgroup analysis between ULR and poor functional outcomes of patients with AIS at 3 months follow-up adjusted for education, SBP, drinking and smoking, TOAST, medical history (AIS or TIA, ICH, hypertension, coronary heart disease, dyslipidemia), in-hospital treatment (antiplatelet, anticoagulant, antihypertensive, antihyperlipidemic, hypoglycemic), and laboratory test (ALB, TC, TG) except the stratified variables. SBP, systolic blood pressure; IS, ischemic stroke; TIA, transient ischemic attack; ICH, intracranial hemorrhage; TOAST, the Trail of Org 10,172 in Acute Stroke Treatment; ALB, albumin; TC, serum total cholesterol; TG, Serum triglyceride; mRS, modified Rankin Scale.

**Figure 5:**
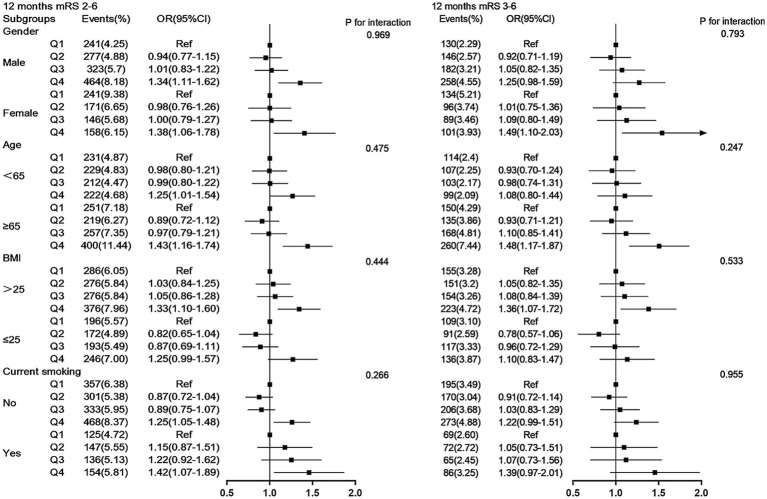
Subgroup analysis between ULR and poor functional outcomes of patients with IS at 12 months follow-up adjusted for education, SBP, drinking and smoking, TOAST, medical history (AIS or TIA, ICH, hypertension, coronary heart disease, dyslipidemia), in-hospital treatment (antiplatelet, anticoagulant, antihypertensive, antihyperlipidemic, hypoglycemic), and laboratory test (ALB, TC, TG) except the stratified variables. SBP, systolic blood pressure; IS, ischemic stroke; TIA, transient ischemic attack; ICH, intracranial hemorrhage; TOAST, the Trail of Org 10,172 in Acute Stroke Treatment; ALB, albumin; TC, Serum total cholesterol; TG, erum triglyceride; mRS, modified Rankin Scale.

At the 3-month follow-up, 2,204 patients (26.74%) had an mRS score of 2–6, and 1,194 patients (14.49%) had an mRS score of 3–6. At the 1-year follow-up, 2021 patients (24.52%) had an mRS score of 2–6, and an mRS score of 3–6 occurred in 1136 patients (13.78%). The associations between ULR and outcomes are presented in Table 2. After adjusting for potential confounding variables, patients with the highest ULR levels significantly had a higher risk of poor outcomes at 3 months, with adjusted OR of 1.33 (95% CI 1.15–1.53) for mRS scores 2–6 and 1.33 (95% CI 1.11–1.59) for mRS score 3–6, compared to the patients with lowest ULR levels. Similar trends were observed for the functional outcomes at 1 year, the adjusted ORs in the fourth quartile group were 1.35 (95% CI 1.16–1.57) and 1.33 (95% CI 1.10–1.60) for mRS scores 2–6 and 3–6, respectively. The trend remained significant even after adjustment for potential confounding factors (P for trend<0.001). The distribution of incidence rate and hazard ratio (HR) or odds ratio of mRS 3–6 was 1.33(95%CI, 1.11–1.59) at 3 months for the Q4 group versus the Q1 group and 1.33(95%CI, 1.10–1.60) at 12 months (Figure 2). Similar results were observed for mRS 2–6 and all-cause mortality at 3 and 12 months (Figure 2; Table 2). Multivariable-adjusted RCS showed a nonlinear relationship between the ULR and poor outcomes. At 3-month and 1-year follow-ups, higher ULR was associated with higher risks of poor outcomes, and mRS scores of 3–6 and 2–6 OR steadily increased, showing J-type association (Figure 3). Subgroup analysis results are shown in Figure 4 and Figure 5, indicating no significant interaction between ULR and the stratified variables (P for interaction > 0.05 for all).

**Table 2:** Association between ULR and clinical outcomes.

Outcome	3 months follow-up			12 months follow-up		
	Events, *n* (%)	Unadjusted	adjusted	Events, *n* (%)	Unadjusted	adjusted
mRS 2 ~ 6	2,204 (26.74)			2021 (24.52)		
Q1	539 (6.54)	Ref	Ref	482 (5.85)	Ref	Ref
Q2	498 (6.04)	0.90 (0.78–1.04)	0.96 (0.83–1.11)	448 (5.44)	0.91 (0.79–1.05)	0.96 (0.82–1.12)
Q3	511 (6.20)	0.93 (0.81–1.07)	1.00 (0.86–1.15)	469 (5.69)	0.96 (0.83–1.12)	1.01 (0.86–1.17)
Q4	656 (7.96)	1.32 (1.15–1.51)	1.33 (1.15–1.53)	622 (7.55)	1.42 (1.23–1.63)	1.35 (1.16–1.57)
P for trend	<0.001	<0.001	<0.001	<0.001	<0.001	<0.001
mRS 3 ~ 6	1,194 (14.49)			1,136 (13.78)		
Q1	290 (3.52)	Ref	Ref	264 (3.20)	Ref	Ref
Q2	242 (2.94)	0.81 (0.68–0.98)	0.87 (0.72–1.05)	242 (2.94)	0.91 (0.75–1.09)	0.96 (0.79–1.17)
Q3	287 (3.48)	0.99 (0.83–1.18)	1.06 (0.88–1.27)	271 (3.29)	1.03 (0.86–1.24)	1.08 (0.89–1.31)
Q4	375 (4.55)	1.36 (1.15–1.61)	1.33 (1.11–1.59)	359 (4.36)	1.44 (1.21–1.71)	1.33 (1.10–1.60)
P for trend	<0.001	<0.001	<0.001	<0.001	<0.001	0.001
All-cause mortality	135 (1.64)			275 (3.34)		
Q1	26 (0.32)	Ref	Ref	47 (0.57)	Ref	Ref
Q2	21 (0.25)	0.81 (0.45–1.43)	0.82 (0.46–1.47)	55 (0.67)	1.17 (0.79–1.72)	1.23 (0.83–1.83)
Q3	29 (0.35)	1.12 (0.66–1.89)	1.17 (0.68–1.99)	59 (0.72)	1.26 (0.86–1.84)	1.30 (0.88–1.92)
Q4	59 (0.72)	2.29 (1.44–3.63)	1.97 (1.22–3.18)	114 (1.38)	2.47 (1.76–3.47)	2.13 (1.50–3.03)
P for trend	<0.001	<0.001	<0.001	<0.001	<0.001	<0.001

Adjusted for age, gender, education, SBP, drinking and smoking, TOAST, medical history (IS or TIA, ICH, hypertension, coronary heart disease, dyslipidemia), NIHSS, in-hospital treatment (antiplatelet, anticoagulant, antihypertensive, antihyperlipidemic, hypoglycemic), and laboratory test (ALB, TC, TG). SBP, systolic blood pressure; mRS, modified Rankin Scale; IS, ischemic stroke; TIA, transient ischemic attack; ICH, intracranial hemorrhage; TOAST, the Trail of Org 10,172 in Acute Stroke Treatment; ALB, albumin; TC, Serum total cholesterol; TG, erum triglyceride; NIHSS, National Institute of Health Stroke Scale.

### 3.3 Comparisons of the associations and prediction between ULR, UA, LY, and clinical outcomes

**Figure 6:**
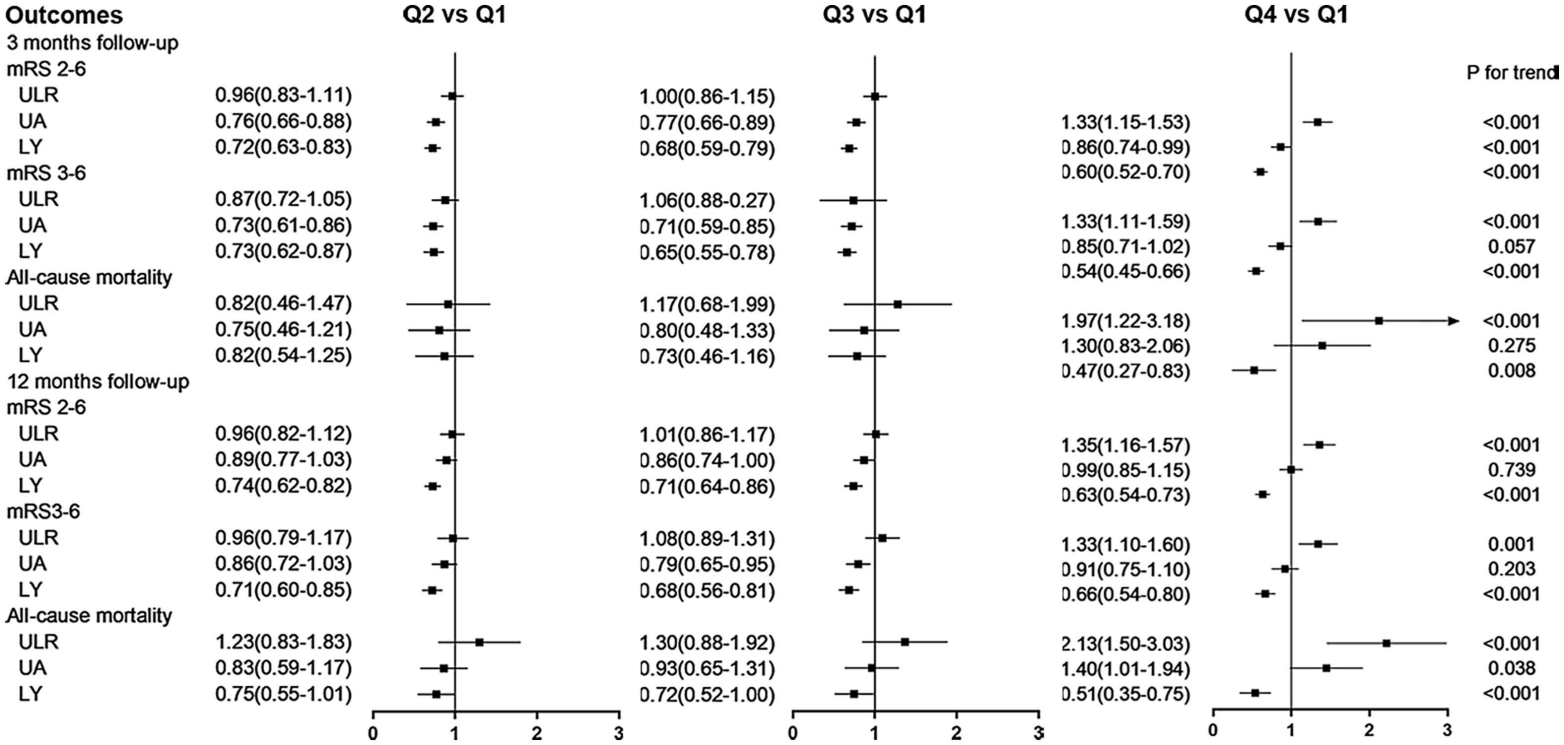
Associations of quartiles of ULR, UA, and LY with clinical outcomes. All models were adjusted for age, gender, education, SBP, drinking and smoking, TOAST, medical history (IS or TIA, ICH, hypertension, coronary heart disease, dyslipidemia), in-hospital treatment (antiplatelet, anticoagulant, antihypertensive, antihyperlipidemic, hypoglycemic), and laboratory test (ALB, TC, TG). SBP, systolic blood pressure; IS, ischemic stroke; TIA, transient ischemic attack; ICH, intracranial hemorrhage; TOAST, the Trail of Org 10,172 in Acute Stroke Treatment; ALB, albumin; TC, serum total cholesterol; TG, Serum triglyceride; mRS, modified Rankin Scale; UA, uric acid; LY, lymphocyte; ULR, uric acid to lymphocyte ratio.

Figure 6 demonstrates the associations of ULR, UA, and LY with clinical outcomes after adjusting for potential confounders. The fourth quartile of UA level was significantly associated with increased risk of all-cause mortality (adjusted OR, 1.40; 95% CI, 1.01–1.94) within 12 months when the first quartile was treated as a reference. The fourth quartile of UA level was significantly associated with increased risk of poor function outcomes (adjusted OR, 0.86; 95% CI, 0.74–0.99 and 0.85;95% CI,0.71–1.02) within 3 months when the first quartile was treated as a reference. The fourth quartile of LY level was negatively associated with all-cause mortality within 3 months (adjusted HR, 0.47; 95% CI, 0.27–0.83) and 1 year (adjusted HR, 0.51; 95% CI, 0.35–0.75), the poor functional outcome at 3 months (adjusted OR, 0.60; 95% CI, 0.52–0.70; adjusted OR, 0.54; 95% CI, 0.45–0.66) and 1 year (adjusted OR, 0.63; 95% CI, 0.54–0.73; adjusted OR, 0.66; 95% CI, 0.54–0.80). Whereas, the fourth quartile of ULR was significantly associated with poor functional outcome and all-cause mortality at 3-month and 1-year follow-up.

We also compared the enhancement in the prediction model for the clinical outcomes by adding ULR, UA, and LY into the basic model. As shown in Table 3, we observed a significant improvement in the predictive performance validated by C-statistic, IDI, and category-free NRI after the addition of ULR into the basic model. The addition of ULR showed more evident predictive power than LY, and UA with the C-statistic (from 0.694 to 0.702; *p* = 0.002), IDI (0.66%; 95%CI, 0.46–0.87; P<0.001), and category-free NRI (18.81%; 95% CI, 12.71–24.91; *p* < 0.001) for the poor outcome of mRS 3–6 at 3 months. A similar trend was demonstrated at 12 months.

**Table 3:** Performance of models to predict clinical outcomes after AIS.

Model	C-statistic		IDI		Category-free NRI	
	Estimate (95%CI)	*p* value	Estimate (95%CI), %	*p* value	Estimate (95%CI), %	*p* value
At 3 months						
mRS 2–6						
Basic model	0.658 (0.645–0.671)	Reference	Reference		Reference	
Basic model+LY	0.662 (0.649–0.675)	0.006	0.31 (0.18–0.44)	<0.001	12.11 (7.71–16.51)	<0.001
Basic model+UA	0.661 (0.647–0.674)	0.060	0.21 (0.10–0.33)	<0.001	8.22 (3.34–13.09)	0.001
Basic model+ULR	0.665 (0.652–0.679)	<0.001	0.66 (0.47–0.84)	<0.001	16.34 (11.71–20.97)	<0.001
mRS 3–6						
Basic model	0.694 (0.678–0.710)	Reference	Reference		Reference	
Basic model+LY	0.699 (0.683–0.714)	0.013	0.30 (0.16–0.45)	<0.001	9.50 (3.47–15.54)	0.003
Basic model+UA	0.697 (0.682–0.713)	0.050	0.24 (0.11–0.38)	<0.001	11.79 (5.67–17.91)	<0.001
Basic model+ULR	0.702 (0.686–0.718)	0.002	0.66 (0.46–0.87)	<0.001	18.81 (12.71–24.91)	<0.001
All-cause mortality						
Basic model	0.781 (0.742–0.819)	Reference	Reference		Reference	
Basic model+LY	0.787 (0.749–0.824)	0.254	0.20 (0.02–0.38)	0.032	12.75 (−3.97–29.46)	0.142
Basic model+UA	0.784 (0.745–0.824)	0.415	0.23 (−0.01–0.46)	0.055	19.68 (2.87–36.48)	0.023
Basic model+ULR	0.797 (0.760–0.833)	0.017	0.29 (−0.10–0.67)	0.014	38.32 (21.33–55.33)	<0.001
At 12 months						
mRS 2–6						
Basic model	0.680 (0.6663–0.6930)	Reference	Reference		Reference	
Basic model+LY	0.683 (0.6702–0.6967)	0.010	0.33 (0.19–0.47)	<0.001	14.21 (9.65–18.77)	<0.001
Basic model+UA	0.680 (0.6670–0.6936)	0.363	0.07 (0–0.13)	0.041	8.235 (3.22–13.25)	0.001
Basic model+ULR	0.689 (0.6712–0.6976)	0.008	0.52 (0.35–0.69)	<0.001	15.34 (10.52–20.15)	<0.001
mRS 3–6						
Basic model	0.721 (0.705–0.737)	Reference	Reference		Reference	
Basic model+LY	0.723 (0.707–0.739)	0.094	0.21 (0.08–0.34)	0.002	10.91 (4.84–16.97)	<0.001
Basic model+UA	0.722 (0.706–0.738)	0.309	0.10 (0.01–0.18)	0.027	7.138 (0.88–13.40)	0.026
Basic model+ULR	0.724 (0.709–0.740)	0.047	0.42 (0.23–0.60)	<0.001	22.74 (16.78–28.70)	<0.001
All-cause mortality						
Basic model	0.760 (0.730–0.790)	Reference	Reference		Reference	
Basic model+LY	0.766 (0.738–0.795)	0.090	0.24 (0.02–0.45)	0.035	9.884 (−2.12–21.89)	0.107
Basic model+UA	0.763 (0.733–0.793)	0.347	0.27 (0.06–0.49)	0.012	15.58 (3.83–27.32)	0.011
Basic model+ULR	0.770 (0.741–0.799)	0.041	0.45 (0.13–0.77)	0.006	29.00 (17.08–40.91)	<0.001

Adjusted for age, gender, education, SBP, drinking and smoking, TOAST, medical history (IS or TIA, ICH, hypertension, coronary heart disease, dyslipidemia), in-hospital treatment (antiplatelet, anticoagulant, antihypertensive, antihyperlipidemic, hypoglycemic), and laboratory test (ALB, TC, TG). UA, uric acid; LY, lymphocyte; ULR, uric acid to lymphocyte ratio; IDI, integrated discrimination improvement; NRI, net reclassification index; SBP, systolic blood pressure; mRS, modified Rankin Scale; AIS, acute ischemic stroke; TIA, transient ischemic attack; ICH, intracranial hemorrhage; TOAST, the Trail of Org 10,172 in Acute Stroke Treatment; ALB, albumin; TC, Serum total cholesterol; TG, Serum triglyceride.; NIHSS, National Institute of Health Stroke Scale.

## 4 Discussion

In the present study, we investigated the relationship between ULR, a newly identified inflammatory biomarker, and stroke prognosis. Our main findings indicate that ULR is positively correlated with poor prognosis and all-cause mortality, even after adjusting for confounding variables. ULR showed numerically better prognostic performance than UA or LY alone. Although the absolute increase in C-statistic was modest (from 0.694 to 0.702 for mRS 3–6 at 3 months), significant improvements in IDI and NRI supported a potential incremental prognostic value of ULR when added to conventional clinical models.

Increasing evidence suggests that post-stroke inflammatory cells play a critical role in the pathological process, such as LY (21), neutrophils (22), leukocytes (23), monocytes (24), T-cells (25) et al. Elevated UA levels and reduced LY counts are commonly considered prognostic indicators in elderly heart failure patients. Wei et al. (26) studied 949 elderly patients with rheumatic heart disease who underwent valve replacement surgery and evaluated the lymphocyte-to-monocyte ratio (LMR), neutrophil-to-lymphocyte ratio (NLR), eosinophil-to-lymphocyte ratio (ELR), basophil-to-lymphocyte ratio (BLR), platelet-to-lymphocyte ratio (PLR), as well as the ULR, found that the ULR, which combines the effects of UA and lymphocyte counts, has a higher predictive value of patient prognosis. Consequently, ULR has been introduced into the research field as a novel biomarker. Yang et al. (18) included 335 patients undergoing thoracoscopic surgical lobectomy for early-stage non-small-cell lung cancer in their study, and demonstrated by propensity score-matched analysis that the ULR can be considered as a novel risk-stratification tool that can be used for prognostic prediction in the postoperative period. Another study, based on a large database from China, which included 93,023 patients without stroke and myocardial infarction and tracked for up to 13 years, demonstrated a significant positive correlation between ULR and stroke risk (27). ULR in stroke patients was positively associated with poor prognosis and all-cause mortality at 3 months and 1 year. This association persisted after adjusting for potential factors. In addition, the addition of ULR to the basic model produced an incremental predictive value for poor prognosis. ULR might be a new biomarker for predicting prognosis in acute ischemic stroke.

The reason why ULR has a better prognostic predictive value than UA and LY can be explained by three possible mechanisms. First, UA has a pro-oxidative stress effect (28). Elevated UA leads to increased oxidative stress, higher plasma renin activity, and activation of systemic inflammatory mediators. This leads to endothelial dysfunction and atherosclerosis, which may contribute to the development of AIS and poor prognosis (29–31). The vascular damage from UA is evident, but its connection to stroke prognosis remains controversial. Liu et al. (32) reported that UA did not significantly predict functional prognosis 3 months after AIS in young patients. Zhong et al. (33) analyzed the data using three methods: cohort study, meta-analysis, and Mendelian randomization study. They found no significant association between UA and prognosis at 3 months post-IS. This could be due to cumulative exposure to UA and its duration over time (34). Second, a systemic inflammatory response, often accompanied by a dramatic decrease in peripheral blood lymphocyte counts, has been implicated in the weakening of the vascular wall (35). LY produce cytokines that induce an inflammatory response post-stroke, associated with thrombosis, brain tissue damage, and neurological deficits (36). Finally, elevated ULR results, due to increased SUA or decreased lymphocyte counts, suggest a strong relationship with high levels of pro-inflammatory mediators and inflammatory responses in patients. UA activates intracellular reactive oxygen species, reactive nitrogen, and Cox-2, generating inflammatory stress. A decrease in lymphocyte counts implies reduced immune self-regulation, exacerbating oxidative stress (37, 38). Third, ULR as a composite marker captures the imbalance between proinflammatory load (elevated UA) and immune regulatory capacity (reduced LY). This imbalance exacerbates ischemia reperfusion injury, propagates neuroinflammation, and impairs neurological recovery. These facts strongly support ULR as a potential new biomarker for predicting the prognosis of AIS.

The main strength of this study lies in its design: a multicenter, prospective registry study featuring a large sample size and long-term follow-up, providing robust support for statistical analysis. Additionally, this study investigates the relationship between the novel biomarker ULR and stroke prognosis, compares the incremental predictive value of UA, LY, and ULR, and conducts subgroup analyses to examine interactions. However, the present study has several limitations. First, this was an observational study and we did not systematically collect detailed data on reperfusion status (e.g., recanalization grade) and concurrent infection (e.g., pneumonia, urinary tract infection) and other unmeasured factors (e.g., preadmission medications). Residual confounding from unmeasured factors cannot be excluded. Second, we only included baseline ULR measurement at admission. Inflammatory and oxidative stress responses after AIS are highly dynamic; single-time-point testing may be affected by sampling time, dehydration, and acute stress, and cannot reflect longitudinal changes. Future studies with serial ULR measurements are needed. Third, ULR was categorized by data-driven quartiles specific to the CNSR-III cohort; we did not determine a clinically applicable cutoff using ROC or Youden index. Finally, all participants were Chinese; generalizability to other ethnicities requires validation.

## 5 Conclusion

In conclusion, we found that elevated ULR levels were associated with poorer functional prognosis and higher risks of all-cause mortality at 3-month and 1-year follow-ups in AIS patients.

## Data Availability

The original contributions presented in the study are included in the article/supplementary material, further inquiries can be directed to the corresponding authors.
